# Linking stochastic assembly to functional potential, redundancy, and trait patterns in bacterial communities

**DOI:** 10.1186/s40168-026-02442-5

**Published:** 2026-06-09

**Authors:** Alizée Le Moigne, Adrian-Ştefan Andrei, Jakob Pernthaler

**Affiliations:** 1 Limnological Station, Department of Plant and Microbial Biology, University of Zurich, Seestrasse 187, Kilchberg, 8802 Switzerland; 2 Centre Eau Terre Environnement, Institut National de La Recherche Scientifique, 490 Rue de La Couronne, Québec City, Québec Canada

**Keywords:** Stochasticity, Functional redundancy, Community assembly, Aquatic bacteria, Functional diversity

## Abstract

**Background:**

Stochastic processes shape the taxonomic composition of microbial assemblages. However, their impact on community functioning remains subject to debate, mainly due to functional redundancy. Little is known on the links between stochasticity and functional redundancy. Here, we assessed how stochastic assembly influences redundancy, functional potential, and trait patterns in twenty parallel lake-water bacterial communities enriched under originally identical conditions. Using gene- and genome-resolved metagenomics, we tested whether incomplete dispersal of genes required for cellobiose uptake and processing—“functional dispersal limitation”—explained variation in cellobiose use.

**Results:**

Several communities were composed of genomes that held the required genes but these communities did not utilize cellobiose, rejecting the notion of “functional dispersal limitation.” We quantified redundancy across major functional categories such as signaling, regulation, and transport. Functional redundancy reflected the stochastic assembly from the total set of genomes. It was lower within than between communities, likely reflecting limiting similarity vs. habitat-driven functional convergence. Category-resolved patterns of functional dissimilarity were conserved across various diversity scales and even across randomly sampled sets of 28,000 bacterial genomes from the Genome Taxonomy Database. Among these categories, functions mediating environmental and microbe-to-microbe interactions and genetic information processing had highest and lowest dissimilarity, respectively. Aquatic bacteria showed the greatest differentiation across most categories.

**Conclusions:**

Stochastic assembly of bacterial communities shaped the functional trait distribution. Functional redundancy inferred from the metagenomes largely reflected the trait patterns of the total set of MAGs. Functional redundancy and dissimilarity varied according to functional category. Comparison with a null model constructed from genomes of the GTDB allowed us to identify functional selection with various strengths according to the functions. While stochasticity diversified community composition, functional patterns remained conserved, reflecting shared ecological and evolutionary constraints tempered by habitat. Hence, using null models as a reference is important to interpret functional redundancy and may provide a more accurate understanding of how stochastic assembly and ecological constraints shape community-level functional organization.

Video Abstract

**Graphical Abstract:**

**Figure:**
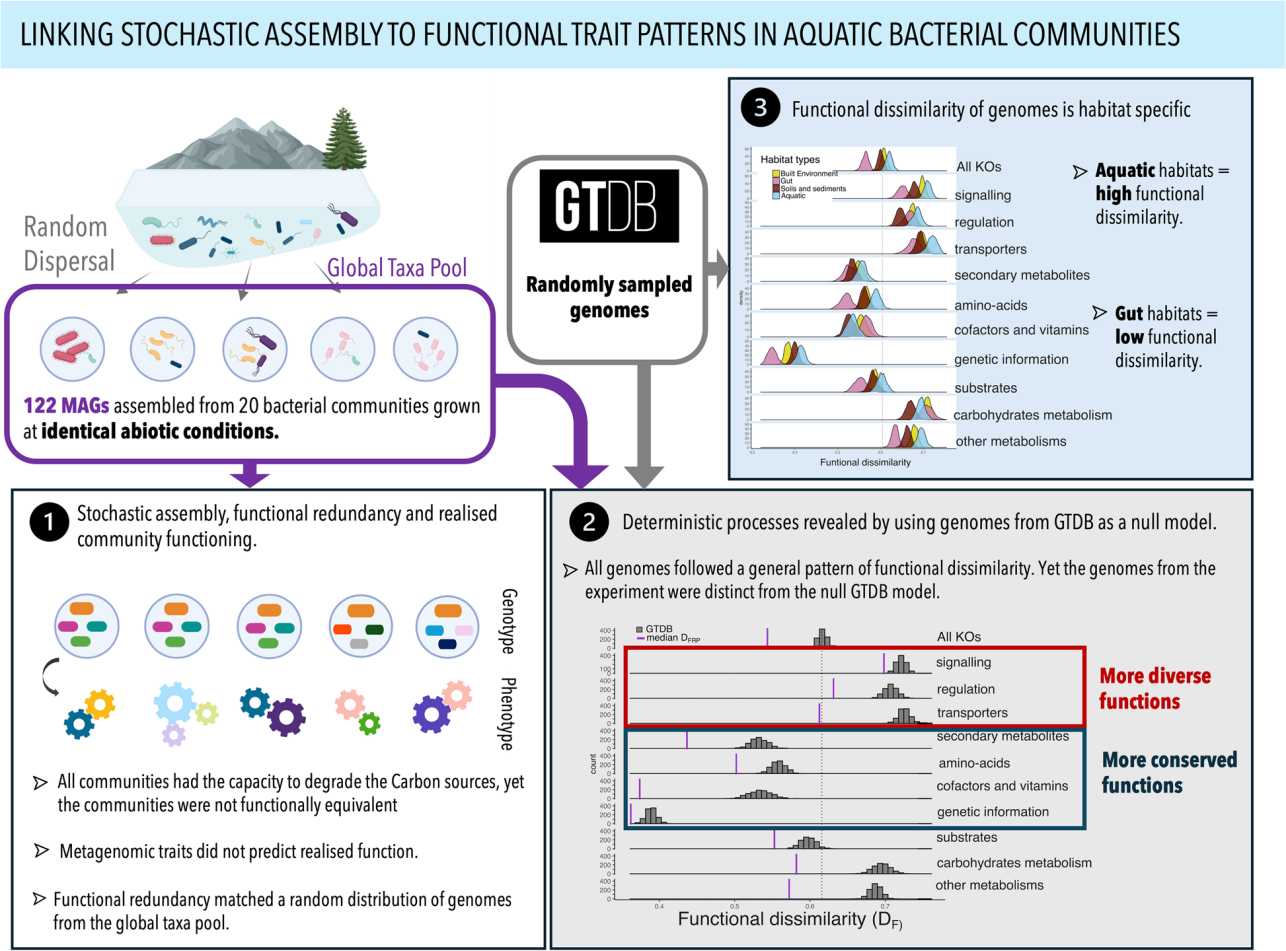

**Supplementary Information:**

The online version contains supplementary material available at 10.1186/s40168-026-02442-5.

## Background

Bacterial communities are affected by stochastic processes, especially when colonizing new habitats [1–3] or recovering from disturbance [4, 5]. The role of stochasticity in defining taxonomic composition is clear; its impact on function is not. Some studies find that functional output is stable despite taxonomic turnover [6–9]. Others report that stochasticity alters both composition and functions [10–14].

Random dispersal, ecological drift, and diversification all drive stochastic assembly [15]. Their functional effects depend on functional redundancy—the degree to which different taxa perform the same role. When redundancy is high, drift or dispersal may shuffle species without altering community functioning. Redundancy varies among clades and traits [16] and between habitats, being, for instance, often lower in host-associated communities than in free-living ones [17]. Rare or keystone functions confined to few or rare taxa may be more easily lost than common functions [18]. Such limits to redundancy can translate stochastic assembly into functional variation, as demonstrated by links between stochasticity and biogeochemical process diversity in agricultural soils [19]. Hence, the balance between stochasticity and redundancy determines when microbial function is predictable and unaffected by stochastic assembly or not.

Functional redundancy can be estimated from phenotypic traits of individual taxa (e.g., Microautoradiography combined with Fluorescence In Situ Hybridisation [20] or Nanoscale Secondary Ion Mass Spectrometer (nanoSIMS) [21]) or inferred from metagenomes, where gene orthologs serve as proxies for metabolic traits [22, 23]. It expresses the fraction of taxonomic diversity not mirrored by functional diversity [24] and can be determined within and between communities [7, 24]. Functional redundancy, akin to species richness, can be measured at various ecological scales. α-Redundancy, the most common scale, captures how functions are organized within communities and how likely a given function is to persist in that community if some species are lost [7]. β-redundancy quantifies the extent to which functional overlap reflects taxonomic similarity between two communities. For instance, β-redundancy would be considered high when two communities express similar functions but dissimilar community composition. β-redundancy can hence reveal the imprint of assembly processes, in particular of selective pressure acting on functional traits that are found in every community in a specific environment irrespective of community composition [24].

We investigated how random dispersal shapes functional redundancy in aquatic bacterial communities grown under identical abiotic conditions. We tested whether dispersal limits the availability of genes for the processing of two substrates with different numbers of potential degrader species in the communities, i.e., with different redundancy with respect to degradation (glucose: high, cellobiose: low). We predicted a “functional dispersal limitation” for cellobiose but not for glucose. We then examined how α- and β-redundancy reflect assembly processes across functional categories. Finally, we compared functional dissimilarity among publicly available genomes from aquatic, soils and sediments, gut, and built environments.

## Methods

### Experimental design and shotgun sequencing

We established twenty replicate bacterial communities in 200 mL microcosms using prefiltered water (0.8-μm pore size polycarbonate filter, Whatman) from the pre-alpine oligo-mesotrophic Lake Zurich (Switzerland, 47° 18′ N, 8° 34′ E) as inocula. Communities were incubated in artificial lake water with glucose and cellobiose for fifteen days at 20 °C in the dark as described in [25]. Experiment 2 from that study was selected for metagenomic analysis due to its high stochastic community assembly, as assessed by 16S rRNA gene resolved taxonomy. At the end of the experiment, 100 mL per microcosm were filtered onto 0.22-μm pore size polyethersulfone filters (GPWP, MF-Millipore) and DNA extracted with the DNeasy PowerBiofilm Kit (Qiagen). Shotgun libraries (paired-end, 150 bp) were sequenced on an Illumina HiSeq 2500 (Novogene) targeting 5 Gbp per microcosm.

### Assembly and binning of microcosm metagenomes

Raw reads were quality-filtered with Trimmomatic v0.36 (Phred = 33, adapter removal; paired-end interleaving) [26]. Additional checks (de novo adapter identification) used “bbmerge.sh” (BBMap v37.02) [27]. Reads were assembled with “metaSPAdes” v3.11.1 [28] (k-mer sizes 21, 33, 55, 77, 99, 127). Curated reads were mapped to contigs > 2.5 Kb with “bbwrap.sh” (BBMap v37.02; kfilter = 31, subfilter = 15, maxindel = 80) per sample. BAM files informed contig abundance profiles via “jgi_summarize_bam_contig_depths” (MetaBAT2 v2.12.1; percentIdentity = 97), followed by binning with MetaBAT2 (default settings) [29]. Bin completeness, contamination, and strain heterogeneity were estimated with CheckM v1.0.3 (“lineage_wf”). [30]. Bins with completeness > 40% and contamination < 5% were retained as Metagenomes Assembled Genomes (MAGs, *n* = 122, median completeness = 94.67%, median contamination = 0.7%).

### Taxonomic annotation and abundance of MAGs

All MAGs (*n* = 122) were taxonomically classified with GTDB-Tk v0.1.6 (default settings) [31]. Relative abundances in their source metagenomes were estimated by competitive recruitment against normalized datasets (33 × 10^6^ reads) using “bbmap.sh” (BBMap v37.02; kfilter = 31, subfilter = 15, maxindel = 80) [27]. Within pre-defined taxonomic groups, population-level clusters shared across communities were identified by hierarchical agglomerative clustering at 99% ANI (average linkage) calculated as described in [32]. MAGs with 99% ANI were pooled as metagenomic OTUs (mOTUs) (Supp. Mat. 1: Fig. S1) and the frequency of their individual traits was averaged for subsequent analyses. MAGs (specific for single microcosms) were used for all analyses of alpha-diversity and on the total pool of taxa, while mOTUs (shared among several microcosms) were used to assess beta-diversity. We also analyzed 28,714 GTDB genomes (release R05-RS95) [33]; quality statistics are available at the GTDB website (https://gtdb.ecogenomic.org/stats/r95).

### Functional annotation of contigs, MAGs, mOTUs, and of GTDB genomes

Contigs, MAGs, mOTUs, and GTDB genomes were subjected to protein-coding gene prediction with Prodigal v2.6.3 (default parameters)) [34], and predicted proteins were assigned to KEGG Orthologues (KOs) using BlastKOALA v2.2 [35]. Contig-level annotations were weighted by contig relative abundance and sequencing depth (average coverage obtained by mapping 20 × 10^6^ reads with BBMap [27]) and then aggregated per microcosm generating a microcosm-by-trait matrix (20 microcosms × 4999 KOs). This contig dataset—being the most complete in terms of the totality of detected genes—was used only for gene-based analyses linking KO profiles to glucose and cellobiose consumption. MAG annotations were aggregated per genome to produce a taxa-by-trait matrix (122 MAGs × 4779 unique KOs). We weighted this taxa-by-trait matrix by the relative abundance of the MAGs obtained by competitive recruitment. For the GTDB genome set, KO profiles were used as a null model of functional gene content in bacterial genomes. To exclude genomes with unusually small or large functional repertoires (often restricted to specific taxa), we fitted a gamma distribution to the number of KOs per genome (X ~ Γ(shape = 3.54, rate = 1.66 × 10^−3^)) and removed genomes in the 2% tails (< 500 or > 5000 KOs). The filtered GTDB set comprised 28,714 genomes, yielding a 28,714 × 8342 taxa-by-trait matrix.

### Functional gene repertoire and observed community functioning

The consumption of both cellobiose and glucose was followed daily in each microcosm during the experiment and is reported in [25], Experiment 2. This experiment also led to the classification of microcosms that were consumers (*n* = 7) or non-consumers (*n* = 13) of cellobiose [25]. We assessed the relationships between observed functional properties and genes using different gene subsets using correlations and Random Forests (R “randomForest”) [36]: Firstly, we focused on 6 genes involved in the early degradation steps of cellobiose (Supp. Mat. 1: Table S1) to evaluate the capacity to consume cellobiose. Secondly, we selected all genes involved in glucose and cellobiose degradation pathways (Supp. Mat. 1: Table S2 category “substrates”) to evaluate the capacity to consume both substrates. Thirdly, we used the whole functional gene repertoire to assess the relationship between the above listed 3 sets of genes (cellobiose, substrates, whole functional gene repertoire, both from the abundance-weighted contig and the abundance-weighted MAG datasets), and 3 independent functional properties: the absolute cellobiose consumption, the classified microcosms (consumers/non-consumers of cellobiose), and the absolute consumption of both, glucose and cellobiose. We included all 20 microcosms when performing gene-based analyses with the contig dataset. By contrast, only 14 microcosms where at least 50% of reads were binned to MAGs were used for the genome-based analysis (i.e., the MAG dataset) to infer community functional properties (Supp. Mat. 1: Fig. S2). Lastly, we assessed the relationship between the taxonomic composition of microcosm communities (using both MAGs and 16S operational taxonomic units (OTUs) from our previous study [25]) and the absolute glucose and cellobiose consumption.

### Definition of functional categories

KEGG Orthology release 114.0 [37] was used to classify KOs into 10 categories. Using keyword/phrase searches (Supp. Mat. 1: Table S2), we grouped KOs into: environment–microbe and microbe-microbe interactions (signalling, transcription regulation, transporters, secondary metabolites); essential metabolism (amino-acid metabolism, cofactors/vitamins, genetic maintenance); genes related to carbohydrates in cultivation medium (glucose/cellobiose metabolism = “substrates”); and other metabolic functions (carbohydrates metabolism, other metabolisms). An “All functional genes” category included all KOs; other categories are its subsets. Gene counts per category are in Supp. Mat. 2 and 3; some KOs belong to multiple categories (Supp. Mat. 1: Fig. S3).

### Functional redundancy and functional dissimilarity metrics

For this analysis, we focused on the 14 microcosms with at least 50% of binned reads in which the MAGs were representative of the overall community (Supp. Mat. 1: Fig S2).

We computed functional β-redundancy (${R}_{\beta }$), i.e., the fraction of taxonomic dissimilarity between two communities that is not expressed by functional dissimilarity, based on the measure described in [24] (Eq. 1).

1 $$\begin{array}{c}{R}_{\beta }= \frac{{(D}_{S\beta }-{D}_{F\beta })}{{D}_{S\beta }}\end{array}$$

where ${R}_{\beta }$ is the functional β-redundancy, ${D}_{S\beta}$ is the taxonomic dissimilarity, and ${D}_{F\beta}$ is the functional dissimilarity between two communities. Taxonomic dissimilarity, ${D}_{S\beta}$ was calculated as the Bray–Curtis dissimilarity (BC) index computed from the relative abundances of mOTUs.

The β functional dissimilarity (${D}_{F\beta }$) was calculated as the algorithmic measure detailed in [24] (Eq. 2).

2 $$\begin{array}{c}{D}_{F\beta }= \sum\limits_{i=1}^{S1}\sum\limits_{j=1}^{S2}{d}_{ij} \times a\left(i,j\right)\end{array}$$

where ${d}_{ij}$ is the functional dissimilarity between mOTU_i_ and mOTU_j_, computed as the BC dissimilarity index calculated from the gene composition of the mOTUs. $a\left(i,j\right)$ is the proportion of mOTU_i_ in community 1 assigned to mOTU_j_ in community 2 in order to minimize the total functional dissimilarity between communities. S1*,* S2 represent the taxon richness in each community, respectively. The computation was performed in R with the function “betaUniqueness” from the package “adiv” [38]. The input matrices were the mOTUs community matrix (70 mOTUs × 14 microcosms) and the mOTUs functional trait matrix (4505 KOs × 70 mOTUs).

Functional α-redundancy was calculated based on the normalized functional redundancy described in [7] (Eq. 3).

3 $$\begin{array}{c}{R}_{\alpha }= \frac{{(D}_{S\alpha }-{D}_{F\alpha })}{{D}_{S\alpha }}\end{array}$$

where ${R}_{\alpha}$ is the functional α-redundancy, ${D}_{S\alpha}$ is the taxonomic diversity, and ${D}_{F\alpha}$ is the functional diversity within the community. The Gini-Simpson index computed on the relative abundances of the MAGs was used as a measure for the taxonomic diversity ${(D}_{S\alpha})$ . The functional α-dissimilarity $({D}_{F\alpha})$ was calculated as Rao’s quadratic entropy (Eq. 4).

4 $$\begin{array}{c}{D}_{F\alpha }= \sum\limits_{i=1}^{S}\sum\limits_{j\ne i}^{S}{d}_{ij} \times {p}_{i}{p}_{j}\end{array}$$

where ${d}_{ij}$ is the functional dissimilarity between MAG_i_ and MAG_j_ computed as the BC dissimilarity index based on the taxa-by-traits matrix and ${p}_{i}$ and ${p}_{j}$ are the relative abundances of MAG_i_ and MAG_j_ respectively. S is the taxon richness of the community. ${D}_{F\alpha }$ was computed with a modified version of the function “rao.diversity” from the package “SYNCSA” [39] where the default Gower index was replaced by the BC dissimilarity (Code provided in Figshare). The input matrices were the MAGs community matrix (88 MAGs × 14 microcosms) and the MAGs functional trait matrix (4495 KOs × 88 MAGs).

We generated null models of functional α- and β-redundancy by shuffling MAG/mOTU identities within community matrices (permutation test, *n* = 999) while fixing relative abundances and richness; this altered gene profiles (affecting ${d}_{ij}$ in Eqs. 2 and 4 and hence ${D}_{F}$) but kept ${D}_{S}$ constant. Deviations from the null were deemed significant at *α* = 0.05. We computed α- and β-redundancy and α- and β-functional dissimilarity for the 10 categories, compared them with Friedman tests on logit-transformed values, contrasted each to “all KOs” via paired Wilcoxon with False Discovery Rates (FDR), method Benjamini-Hochberg (BH) using microcosm identity for ${R}_{\alpha }$ and ${D}_{F\alpha }$ and microcosm-pair identity for ${R}_{\beta }$ and ${D}_{F\beta }$ as within-group variable, and reported the effect size as Kendall’s W. Differences between α- and β-redundancy across the categories were tested using Mann–Whitney with FDR (BH) and rstatix’s “wilcox_effsize”. Finally, we computed functional dissimilarity between all MAGs found in the 14 experimental microcosms ${D}_{FTP88}$ (Bray–Curtis functional dissimilarity of the Total Pool of 88 MAGs × 4495 KOs), tested category differences with Friedman on p.beta-rescaled (]0,1[) and logit-transformed dissimilarities using MAG-pair identity as within-group variable, and compared “All functional genes” to other categories by FDR (BH)-corrected Wilcoxon. Detailed results are given in Supp. Mat. 2.

### Redundancy based on Enzyme Commission (EC) numbers

To avoid underestimating redundancy due to the ortholog nature of KOs, as opposed to “functionally equivalent genes” (e.g., 7 KOs catalyze the reaction from β-D-glucose to β-D-glucose-6P), we performed a complementary analysis by collapsing KOs into EC numbers. KOs catalyzing the same reaction were assigned a unique EC number based on KEGG EC annotations. Out of the 13 221 KOs from the KEGG database, 5644 had a corresponding complete EC number (42.6%). This percentage varied greatly between the functional categories we investigated (Supp. Mat. 1: Fig. S4A). We calculated an EC-based α- and β-redundancy following the methods described in the preceding section and provided the results in Supp. Mat. 1: Fig. S4B for information purposes. Given that the EC-based annotation was less comprehensive than the KO-based one, we focused our study on the latter.

### Redundancy and phylogenetic relatedness

We assessed phylogenetic relatedness effects on functional redundancy between communities by scanning translated MAG proteins with HMMER “hmmscan” v3.1b2 (*E*-value threshold 10^–10^) against TIGRFAMs v15.0. We extracted 118 ubiquitous single-copy markers [33], built phylogeny-aware multiple sequence alignments (MSAs) with PASTA v1.8.3 (default settings) [40], trimmed alignments with BMGE v1.12 (-g 0.5), concatenated MSAs, and inferred phylogenies with IQ-TREE v1.6.10 (-m LG + C10 + F + R10) by applying an LG amino-acid exchange rate matrix for 10 classes plus 11th class of empirical amino-acid profile (counted from the data) and a FreeRate heterogeneity model. The β-Mean Nearest Taxon Distance (β-MNTD) served as a measure of phylogenetic relatedness between microcosms. β-MNTD was calculated with the package “Picante” [41] using code from [42], and correlated with R_β_ (Pearson).

### Functional dissimilarity of GTDB genomes

We compared the functional dissimilarity of all the MAGs from our experiment ${(D}_{FTP122})$ with the functional dissimilarity of a set of publicly available genomes. We randomly subsampled 122 genomes 999 times from the 28 714 × 8 342 taxa-by-traits matrix generated with genomes from the GTDB and calculated the functional dissimilarity between genomes for each of the 999 sets (BC dissimilarity, ${D}_{GTDB}$). We computed the median dissimilarity for each GTDB set and compared it to the median of the 122 MAGs from the experiment. The *p*-value of the difference was calculated as a one-tail threshold of *α* = 0.05. The effect size (ES) was calculated as Eq. 5.

5 $$\begin{array}{c}ES=\frac{(\mathrm{median}\,{D}_{FTP122}-\mathrm{median}\,{D}_{GTDB})}{{IQR}_{GTDB}}\end{array}$$

With ${IQR}_{GTDB}$ as the Interquartile range of the median dissimilarities from the genomes from GTDB.

### Habitat type specificity of functional dissimilarity

We randomly sampled 906 GTDB genomes from each of four habitat types (Aquatic, Soils and Sediments, Built Environment, and Gut) using semi-manual BioSample curation of the metadata contained in the assembly reports of the NCBI (ftp://ftp.ncbi.nlm.nih.gov/genomes/ASSEMBLY_REPORTS/assembly_summary_genbank.txt). The isolation environment of genomes varied within habitats: Aquatic habitat contained genomes from seawater (30%), surface freshwater (36%), freshwater ground water and subsurface seawater (21%); Soils and Sediments contained soils from the Arctic permafrost to temperate forests (52%), sediments both from the ocean and lakes (36%) and muds (7%); Built Environment was mostly represented by genomes originating from wastewater treatment infrastructures (60%), bioreactors (11%), metal and plastic surfaces (10%), and hospital surfaces (4%). Gut habitats contained human (64%) and animal gut samples (25%, including mammals, invertebrates, and birds). All 3624 genomes were of high-quality. “Built environment”, “Soils and sediments”, and “Aquatic” had comparable diversity at various taxonomic levels, while genomes from “Gut” had reduced diversity (Supp. Mat. 1: Fig. S5). We randomly subsampled 100 genomes 999 times for each habitat type and computed genomic functional dissimilarity, (calculated as the BC dissimilarity from taxa-by-traits matrix) and the median functional dissimilarity for each randomization for each category. We tested effects of category and habitat on functional dissimilarity with a beta regression (“betareg”), conducted post hoc Estimated Marginal Means analyses and adjusted *p*-values by Tukey’s method (detailed results in Supp. Mat. 3).

## Results

### Community taxonomic and functional composition

**Fig. 1:**
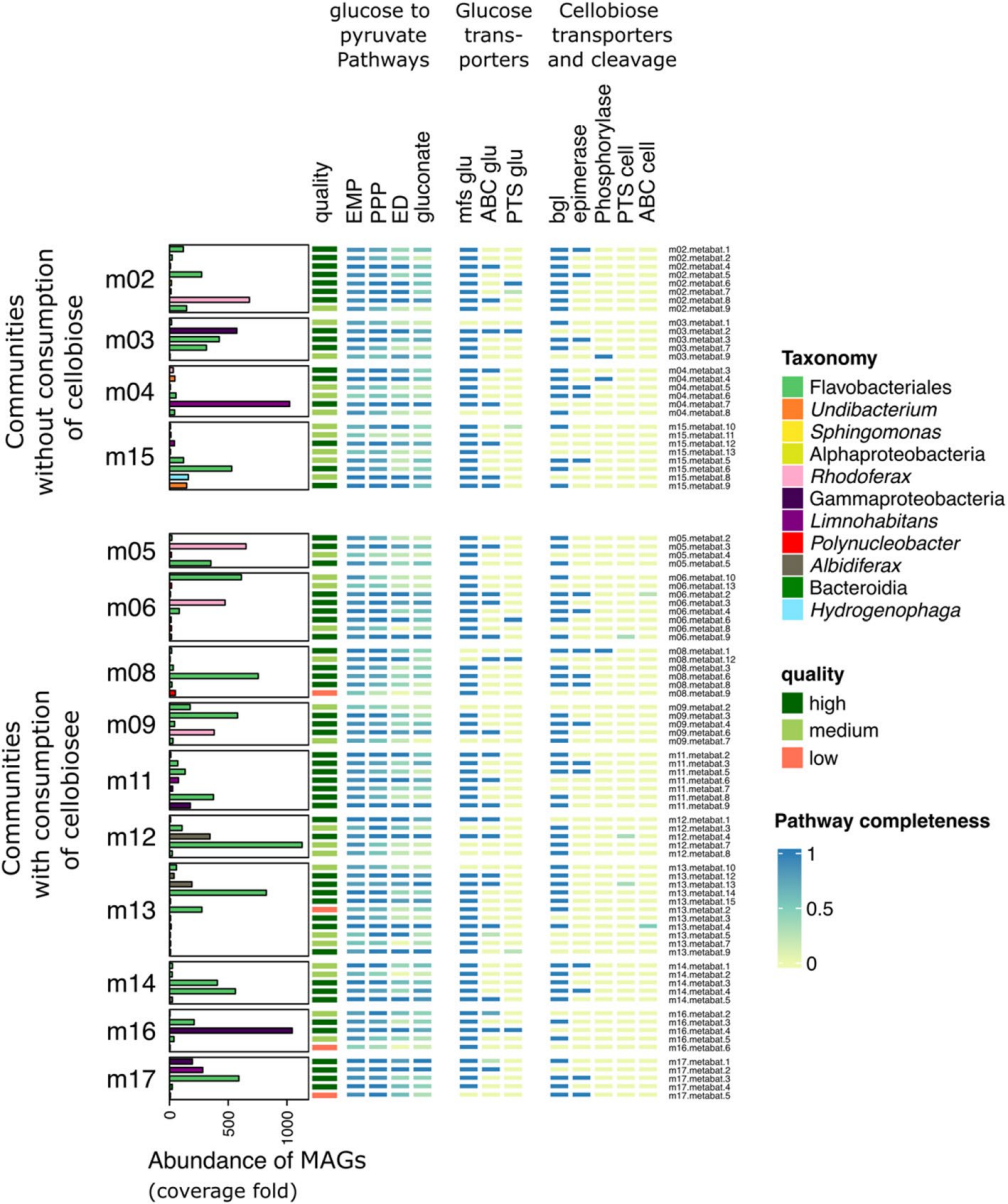
Taxonomic and functional composition of the experimental communities. Bar graph: composition and relative abundances of MAGs in microcosms with and without cellobiose consumption. Heatmap: first column: MAG quality, other columns: completeness of pathways or proteins involved in the degradation of glucose and cellobiose. EMP: Embden-Meyerhof-Parnas, PPP: Pentose Phosphate Pathway, ED: Entner-Doudoroff, gluconate: glycolysis via gluconate, bgl: beta-glucosidases, glu: glucose, cell: cellobiose. *n* = 14 microcosms

We recovered 122 MAGs from the 20 microcosms (74 high-quality, 40 medium-quality, 8 low-quality). MAG completeness ranged 40.8–99.9% (median 94.7%) and contamination 0–4.6% (median 0.7%; Supp. Mat. 1: Fig. S6A). Taxonomy matched previous results from 16S rRNA gene sequencing [25], with a dominance of *Flavobacterium* (53 MAGs) and high abundance of *Rhodoferax* and other *gammaproteobacteria* (Fig. 1). MAG richness per microcosm was 4–11 (mean 6; Fig. 1, Supp. Mat. 1: Fig. S2), approximately 1/6 of OTU richness (6 ± 2 vs. 40 ± 9 OTUs). Most reads were binned in 14 of 20 communities (Supp. Mat 1: Fig. S2), capturing abundant taxa.

Functional annotation of the 122 MAGs yielded 4779 genes, averaging 1,416 ± 412 per MAG (min 638, max 2,557). Thus, 94.6% of the totality of genes annotated in all contigs prior to MAG assembly (*n* = 4999) were also present in the MAGs. Per microcosm, 92.5% ± 0.8% of MAG genes mapped to the 10 predefined functional categories (Supp. Mat. 1: Fig. S6B).

### No “functional dispersal limitation” of substrate degradation genes

**Fig. 2:**
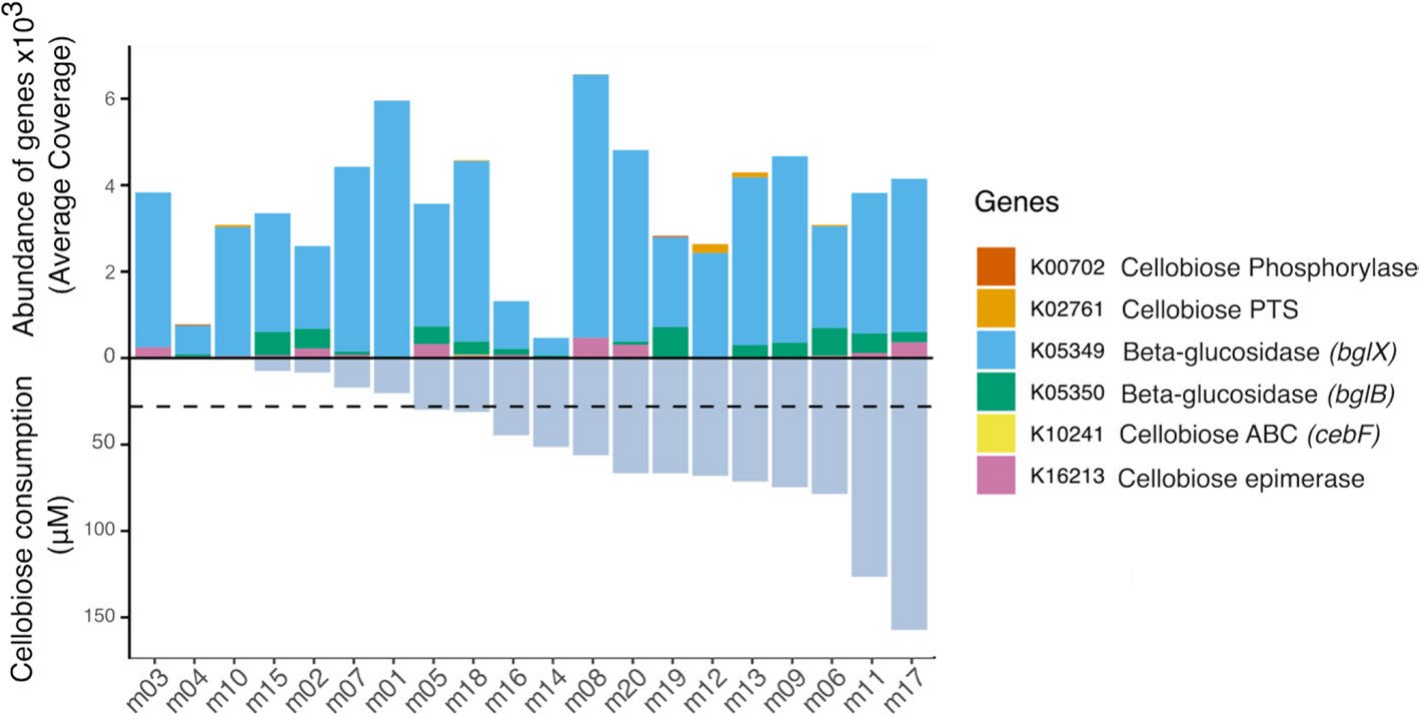
Lack of correspondence between the presence and abundance of genes (abundance-based contig dataset) involved in cellobiose degradation in all 20 microcosms and observed cellobiose consumption. Dotted line: empirical lower threshold above which cellobiose consumption was deemed significant (34.2 μM, i.e., 20% of the concentration at the beginning of the experiment). Cellobiose consumption data and its threshold value are reported from [25]

Seven of 20 communities did not degrade cellobiose (Fig. 2; data from Table 2, E2 in [25]). We hypothesized that the absence of rare degraders (and their genes) due to random dispersal was responsible for the absence of cellobiose degradation and thus examined genes for glucose and cellobiose. One hundred one such genes were found in 88 MAGs (14 microcosms), averaging 2.1% ± 0.13 of annotated genes with low between-community variability (Supp. Mat. 1: Fig. S6B). Of the 14 microcosms well-represented by MAGs, 2 did not consume glucose and 4 did not consume cellobiose; yet every community had at least one MAG with glucose-processing genes and at least one with cellobiose uptake/degradation genes (Fig. 1), indicating that the absence of consumption was not caused by the lack of the necessary genes.

### Functional gene repertoire does not predict substrate consumption

We tested whether gene repertoires would classify cellobiose-consuming microcosms and non-consumers. Six early-step cellobiose genes (transport/cleavage) were present across microcosms (Figs. 1 and 2); beta-glucosidases (bglX, bglB) and a cellobiose epimerase were most common. Total abundance of the six genes did not correlate with cellobiose consumption (Pearson; gene-based: *n* = 20, df = 18, *t* = 0.73, *p* = 0.48, *r* = 0.17; genome-based: *n* = 14, df = 12, *t* = 0.99, *p* = 0.34, *r* = 0.27; Fig. 2). Random forests failed to classify consumers (gene-based: *n* = 20, 500 trees, OOB = 70%; genome-based: *n* = 14, 500 trees, OOB = 50%).

Next, we analyzed the category “substrate” genes (glucose and cellobiose). Classification remained poor (RF; gene-based: *n* = 20, 106 genes, 500 trees, OOB = 40%; genome-based: *n* = 14, 101 genes, 500 trees, OOB = 36%). Distances based on absolute substrate consumption (Euclidean) and substrate gene repertoires (Bray–Curtis) were uncorrelated (Mantel, Pearson, 999 permutations; gene-based: *r* = − 0.19, *p* = 0.98; genome-based: *r* = 0.02, *p* = 0.41).

As substrate degradation did not track substrate traits, we tested the full functional repertoire. Random forests again failed to classify cellobiose consumers and non-consumers (gene-based: 4999 genes, 1500 trees, OOB = 50%; genome-based: 4495 genes, 1500 trees, OOB = 35.71%). Taxonomy performed no better to explain substrate degradation (MAGs: *n* = 14, 500 trees, OOB = 28.57%; 16S: *n* = 20, 500 trees, OOB = 40%).

### Functional gene distribution indicates stochastic assembly from the total pool of taxa

To infer assembly processes, we analyzed the 14 microcosms well-represented by MAGs (Supp. Mat. 1: Fig. S2). Proportions of genes in each functional category were highly similar (mean Bray–Curtis 0.05 ± 0.03; range 0.01–0.10; *n* = 14; Supp. Mat. 1: Fig. S6B). KO-level dissimilarity was modest (mean Bray–Curtis 0.32 ± 0.08; range 0.17–0.57; *n* = 14), suggesting weak homogeneous selection.

**Fig. 3:**
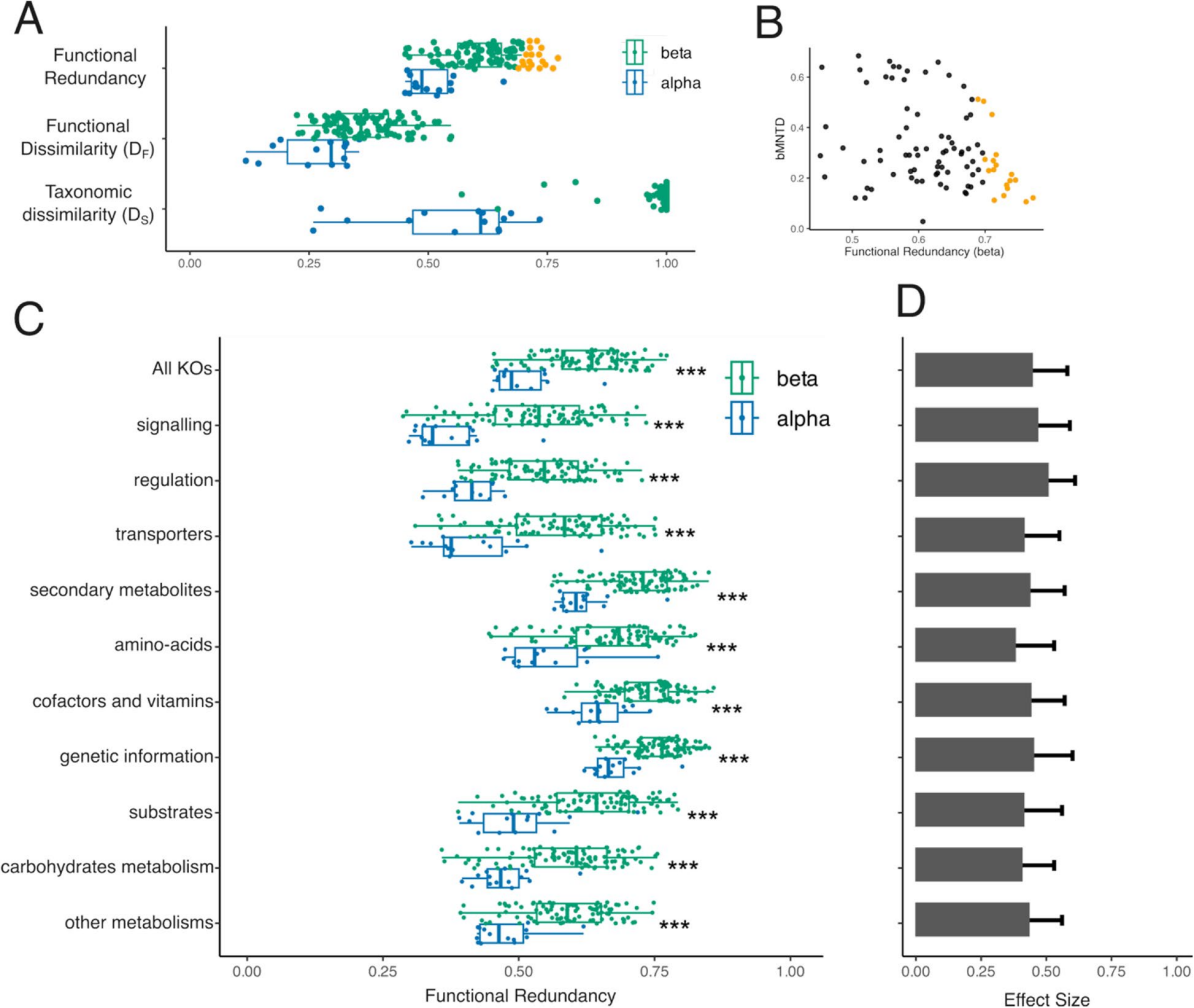
Functional redundancy across diversity scales and various functional categories. **A** α- and β-functional redundancy and their components functional dissimilarity (*D*_F_) and taxonomic dissimilarity (*D*_S_) as calculated from all KOs. Pairs of communities with a higher-than-expected β-functional redundancy are highlighted in orange. **B** Correlation between β-functional redundancy and phylogenetic relatedness (β-MNTD) (Pearson: *r* = −0.32, *p* = 0.0017), the same pairs as in A are highlighted in orange. **C** Functional redundancy for various functional categories. Asterisks: significant difference between α- and β-functional redundancy at *p* < 0.001. **D** Effect size of the difference between α- and β-functional redundancy (error bars: 95% confidence Intervals)

Considering ß-taxonomic dissimilarity (mean D_Sβ_: 0.98 ± 0.07; range 0.57–1; *n* = 14; Fig. 3A) and functional dissimilarity (mean D_Fβ_: 0.36 ± 0.07; range 0.22–0.55; *n* = 14), mean between-community redundancy R_β_ (“all KOs”) was 0.63 ± 0.08 (Fig. 3A). Under mOTU shuffling, 80.2% of pairs (*n* = 73) matched the null; 19.7% (*n* = 18) showed higher-than-null R_β_ (Fig. 3A; Supp. Mat 1: Fig. S7) and were phylogenetically similar (Fig. 3B).

Alpha taxonomic diversity (Gini–Simpson) averaged 0.54 ± 0.15 (range 0.26–0.73); functional dissimilarity averaged 0.26 ± 0.08 (range 0.12–0.35; Fig. 3A). Mean within-community redundancy R_α_ (“all KOs”) was 0.51 ± 0.06 (Fig. 3A). After MAG shuffling, no community differed from the null (Supp. Mat. 1: Fig. S8), implying no detectable competitive exclusion or facilitation. One microcosm (m14) had relatively high R_α_ (not above null), comprising four *Flavobacteria* and one *Albidiferax*. Across categories, R_β_ always exceeded R_α_ with little effect-size variation (Fig. 3C, D; Supp. Mat. 2).

The complementary redundancy analysis based on EC numbers led to higher redundancy values (R_α__KO = 0.51 ± 0.06 vs. R_α__EC = 0.59 ± 0.04, *t*-test *t* = −5.9, df = 20, *p* = 7 × 10^–6^; and R_β__KO = 0.63 ± 0.08 vs. R_β__EC = 0.68 ± 0.06, *t*-test *t* = −5.9, df = 174, *p* = 1.3 × 10^–8^, category “All genes”). The differences in redundancy between categories were similar to the pattern observed in the analysis realized with KOs with exceptions for the categories “signalling” and “transporters” (Suppl. Mat. 1: Fig. S4B), where only a small portion of KOs had EC equivalent (signalling = 32% and transporters = 10%).

### A universal pattern of category-resolved functional dissimilarity

**Fig. 4:**
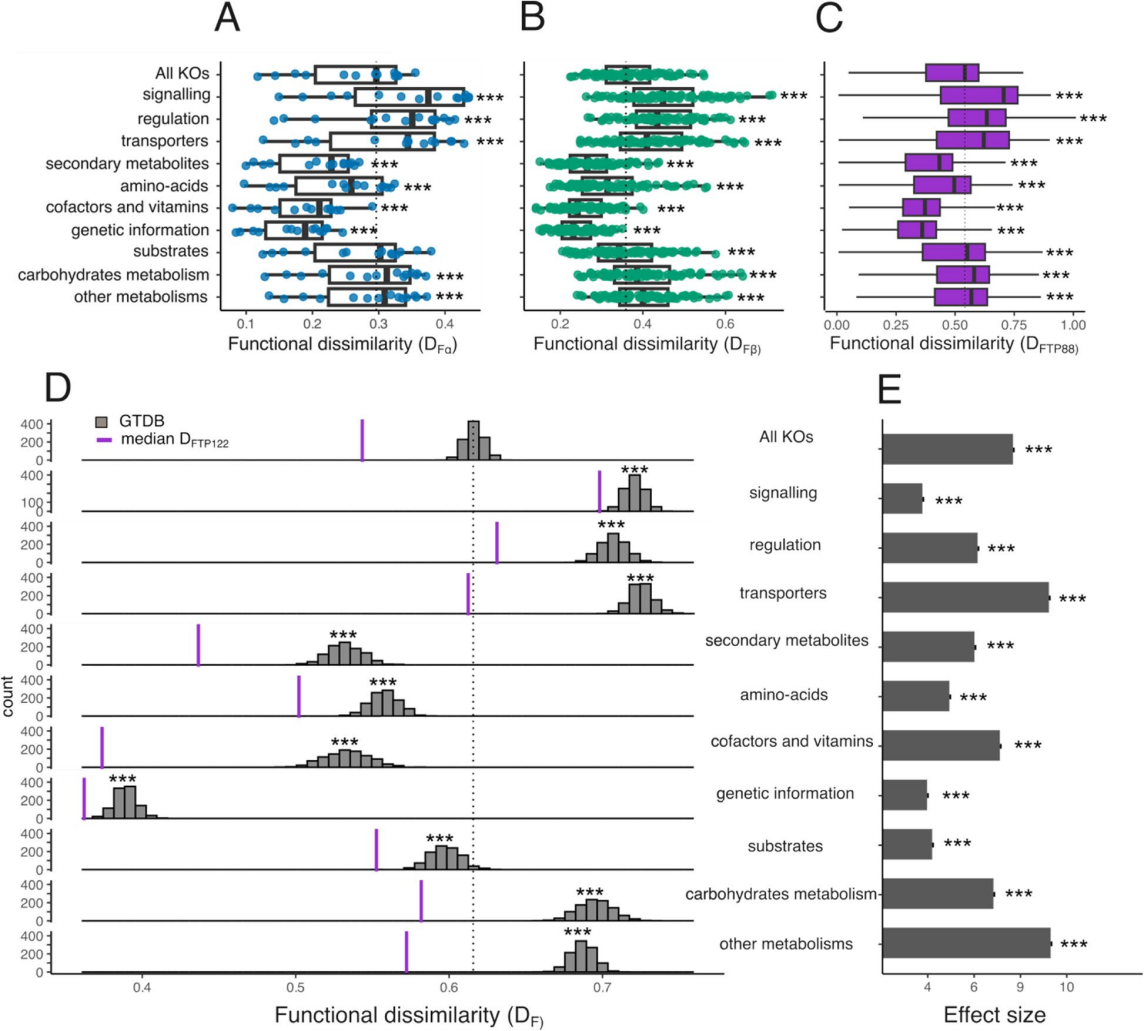
**A**–**C** Dissimilarity patterns of MAGs from experimental communities in functional categories: **A** α-functional dissimilarity, **B** β-functional dissimilarity and **C** functional dissimilarity of the total species pool (all 122 MAGs from experimental communities). **D** Comparison of the functional dissimilarity of MAGs from experimental communities with that of 122 randomly sampled GTDB genomes (999 draws). **A**–**D**: asterisks: significant difference to category “all KOs” (*p* < 0.001); dashed line: median functional dissimilarity of category “all KOs”. **E** Effect size and significance (asterisks, *p* < 0.001) of the difference between the median functional dissimilarities of MAGs from experimental communities and of GTDB genomes

Because R_α_ and R_β_ largely reflected stochastic assembly from the total pool of taxa investigated, we examined functional dissimilarities among the 88 MAGs from this total pool in the 14 microcosm. The α (D_Fα_), β (D_Fβ_) and total pool of taxa (D_FTP88_) functional dissimilarities showed highly similar patterns across the categories (Fig. 4A–C). We hypothesized that this category-level dissimilarity patterns were general features of bacterial genomes and analyzed random sets of GTDB genomes. Signalling, transcription regulation, and transporters were more dissimilar than the “all KO” baseline; secondary metabolites, amino-acid metabolism, cofactors and vitamins metabolism, and genetic information maintenance were more similar (Friedman: D_Fα_: *χ*^2^ = 129, df = 10, *p* < 2.2 ×10^–16^, D_Fβ_: *χ*^2^ = 836, df = 10, *p* < 2.2.10^–16^, D_FTP88_: *χ*^2^ = 28 614, df = 10, *p* < 2.2.10^–16^, D_GTDB_: *χ*^2^ = 2.57 × 10^6^, df = 9, *p* < 2.2.10^–16^, Supp. Mat. 2). Sampling more genomes reduced variability but not the median dissimilarity (Supp. Mat. 1: Fig. S9).

### Functional repertoire of experimental metacommunity reflects environmental selection

While there was no evidence of functional distinction within or among microcosms (Figs. 3A and 4A, B), we hypothesized that the total set of 122 MAGs that dominated after enrichment should as a whole be a product of abiotic or biotic selection. As expected, the functional dissimilarity of our MAGs was consistently lower than that of random sets of 122 genomes from GTDB, indicating species sorting and/or a common habitat of origin (Fig. 4D). More importantly, the extent of deviation from the null model (distance to median, effect size) provided information on the specificity of the trait distribution of MAGs from our experiment: The largest difference between our MAGs and the GTDB null (median) was in “Cofactors and vitamins” and the highest effect sizes were in “Other metabolism” and “Transporters” (Fig. 4E). This suggests that the (biotic and abiotic) selection pressure of our setup most strongly affected these specific trait categories.

### Habitat-specific functional dissimilarity patterns

**Fig. 5:**
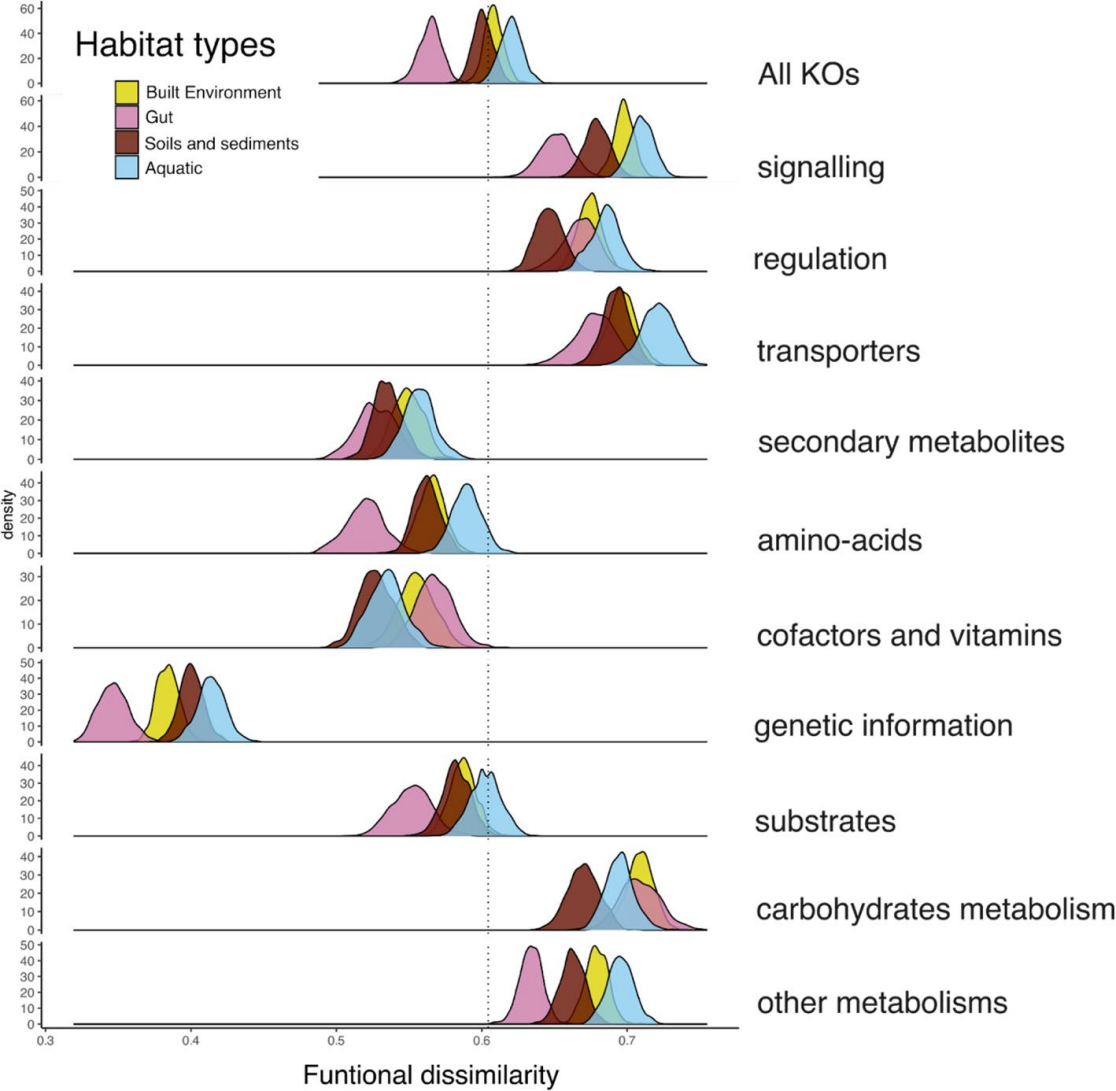
Distributions of functional dissimilarity of 3624 randomly chosen GTDB genomes from different habitat types (999 draws of 100 genomes from each habitat). The dashed line depicts the median functional dissimilarity of all genes (category “all KOs”) of genomes from all habitats. The corresponding statistical details are in Supp. Mat. 3

GTDB genome sets from different environments generally followed the above described category-resolved dissimilarity pattern (Fig. 5; analysis of deviance from the beta regression model: functional category, df = 10, *χ*^2^ = 3,749,807, *p* < 2.2 10^–16^, Effect size (Phi) = 9.24), except for Gut genomes, which were more dissimilar than their “all KOs” baseline in “Cofactors and vitamins” (Fig. 5; Supp. Mat. 3). There were significant habitat effects (Habitat, df = 3, *χ*^2^ = 10,138, *p* < 2.2 10^–16^, Effect size (Phi) = 1.52): aquatic genomes had the highest functional dissimilarity when all genes were considered and in 8/10 categories, whereas gut genomes were most similar in 7/10 categories (Fig. 5; Supp. Mat. 3). The interaction was also significant but with a smaller effect (Functional category × Habitat, df = 30, *χ*^2^ = 60,885, *p* < 2.2 10^–16^, effect size (Phi) = 1.18).

## Discussion

### Metagenomic “functional potential” did not capture functional reality

Metagenomics links genes to ecosystem functions [43, 44]. Gene inventories are often used as trait proxies [45], yet phenotype depends on realized rather than fundamental niche [46, 47]. Using parallel communities developing on two closely related carbon sources, we expected a clear relationship between metagenomic traits and realized function (cellobiose degradation), possibly stronger than between function and taxonomic composition [25]. Instead, neither functional gene analyses (Fig. 2) nor Random Forest models distinguished communities that degraded cellobiose from those that did not based on their gene repertoire. All microcosms possessed genes for glucose and cellobiose degradation (Fig. 1), refuting our hypothesis of “functional dispersal limitation” [48]. Suppression of β-glucosidase expression by glucose [49] could explain part of the variation, but glucose depletion did not consistently trigger cellobiose use [25]. Our findings point to additional determinants of realized function invisible to metagenomics that could have regulated the expression of those genes, such as taxon-specific gene regulation [50], antagonistic inhibition [51], or higher-order species interactions [52]. The decoupling between genetic traits and realized function underscores challenges for data-driven design of microbial consortia [53].

### Stochastic assembly reflected in the distribution of genes from the total pool of taxa

Functional redundancy can uncouple composition and function in artificial [54, 55] and natural [9, 56] systems. Taxonomically, our microcosms assembled stochastically [25], so we expected high-functional β-redundancy under shared abiotic selection [24]. Only a few pairs exceeded the null redundancy model (Fig. 3A), and these were mostly phylogenetically close (low β-MNTD; Fig. 3B), leaving the scale of selection (gene or genome) unresolved. No deviations appeared in α-redundancy [7], and we saw no dominance of either competition or facilitation. Instead, both metrics pointed to stochastic draws from the total pool of taxa.

Functional categories differed in their redundancy patterns (Fig. 3C). β-redundancy of category “Substrates” resembled the one of “all KOs,” indicating no selection on the substrate degradation genes. However, the definition of functional units could matter, as distinct orthologs may perform identical functions [57], e.g., seven KOs catalyze the reaction from β-D-glucose to β-D-glucose-6P leading to an underestimation of redundancy. To avoid such caveats, we performed an EC-based complementary analysis in which the β-redundancy of category “Substrates” also resembled the one of all EC numbers (“All KO”) (Supp. Mat. 1: Fig. S4), thus consolidating the absence of selection on the substrate degradation genes.

β-Redundancy always exceeded α-redundancy (Fig. 3C), consistent with broader gene sharing among communities experiencing a similar abiotic context than within, where competition may limit functional overlap. The elevated β taxonomic diversity also partly explains the elevated redundancy. Higher β- than α-redundancy was also reported in aquatic macroinvertebrate [58] and rotifer [59] metacommunities.

### Dissimilarity patterns revealed deterministic signals in the total gene pool

Despite varying averages, functional dissimilarity across categories was highly consistent across α-, β-, and total pool of taxa scales (Fig. 4A–C). Genes involved in signalling, regulation, and transport were more dissimilar, while those related to core metabolism or genetic information were less so. This pattern, also found in random GTDB genomes independent of category size or sample size (Fig. 4D, Supp. Mat. 1: Fig. S9), likely reflects plausible evolutionary constraints: fundamental cellular processes are conserved [60, 61], while environmental response traits evolve more freely [22, 62, 63].

MAGs from our experiment consistently showed lower functional dissimilarity than equally sized random GTDB sets (Fig. 4D, E), indicating deterministic selection and shared metacommunity origin. This agrees with global patterns where metagenomic traits distinguish among marine zones [64], soil biomes [43] or between terrestrial and aquatic environments [65]. Differences were strongest for transporters, cofactors and vitamins, and carbohydrate metabolism (Fig. 4E). The vitamin-free medium likely selected for prototrophs, while a phylogenetic signal (enrichment of copiotrophic *Flavobacteria*) contributed to convergence in transporter and carbohydrate metabolism genes [66]. Comparing observed patterns with the GTDB null model was therefore essential in inferring deterministic influences: similar absolute dissimilarities, as in “Genetic information” versus “Cofactors and vitamins,” represented distinct ecological meanings (evolutionary conservation vs. environmental selection) depending on their divergence from random expectations (Fig. 4E).

### GTDB genomes from aquatic habitats displayed higher overall functional diversity

Sorting GTDB genomes by habitat revealed that subsets of aquatic bacteria were the most diverse across functional categories (Fig. 5), providing a functional analogue to the “Paradox of the plankton” concept [67]. The large environmental heterogeneity of aquatic environments could partly explain this higher dissimilarity, e.g., consortia of particle-attached aquatic bacteria show functional heterogeneity at small spatial scales [48, 68]. However, soils and sediments also encompass large environmental diversity, not fully accounting for the high functional dissimilarity of aquatic habitat. On the other end of the spectrum, gut genomes were more functionally similar in most functional categories, consistent with host-imposed constraints [69] and narrower phylogenetic range (Supp. Mat. 1: Fig. S5) that could have restricted functional dissimilarity. Nonetheless, gut genomes were the most functionally dissimilar for the category “cofactors and vitamins” and more dissimilar than aquatic genomes in the category “carbohydrates metabolism,” displaying an interesting case of functional diversity in phylogenetically close genomes compared to aquatic genomes. The intriguing differences in functional dissimilarity among sets of microbes from specific habitats might thus merit further investigation, e.g., in the context of limiting similarity, facilitation and the Black Queen mechanism [70].

## Conclusions

Parallel communities assembled under initial identical abiotic conditions displayed high redundancy of metagenomic traits related to both carbon sources, yet gene presence did not predict functional differences. These findings reveal fundamental limits of the “metabolic potential” concept as an indicator of microbial activity. Metaproteomics, albeit demanding, may better link genetic traits to realized functions [71].

Two null-model approaches exposed complementary assembly processes: random dispersal within the regional pool, but also that genomes in experimental enrichments had deterministic traits, i.e., were less diversified in some categories than global references. Core informational and metabolic genes were generally most conserved, whereas genes for regulation, transport, and environmental interaction were more dissimilar (Fig. 4D). This pattern was slightly modulated by habitat-specific characteristics (Fig. 5). Altogether, our findings underscore the limits of metagenomics to reveal realized community function, and the potential of GTDB-based null models to identify deterministic traits in specific communities.

## Supplementary Information

Supplementary Material 1.

Supplementary Material 2.

Supplementary Material 3.

## Data Availability

The accession number of all used public genomes, taxa-by-traits matrices, and R code are available in Figshare (https://doi.org/10.6084/m9.figshare.c.6734349). The sequences, contigs, and MAGs were deposited at ENA under the accession number PRJEB65858.
