# Supplementary material for: Linking stochastic assembly to functional potential, redundancy, and trait patterns in bacterial communities
Source: Microbiome. 2026 Jun 9;14:201. doi: 10.1186/s40168-026-02442-5 (PMC13471509; doi:10.1186/s40168-026-02442-5)
Supplement: Supplementary file 1 [file 40168_2026_2442_MOESM1_ESM.pdf]

Supplementary Material

Linking stochastic assembly to functional potential, redundancy and trait patterns in bacterial communities

Authors

Alizée Le Moigne[1,2*], Adrian-Stefan Andrei[1], Jakob Pernthaler[1]

[1]Limnological Station, Department of Plant and Microbial Biology, University of Zurich, Seestrasse 187, 8802 Kilchberg, Switzerland

[2]Institut National de la Recherche Scientifique, Centre Eau Terre Environnement, 490 rue de la Couronne, Québec city, Québec, Canada

*Corresponding author: Alizée Le Moigne

Email: alizee.le-moigne@inrs.ca

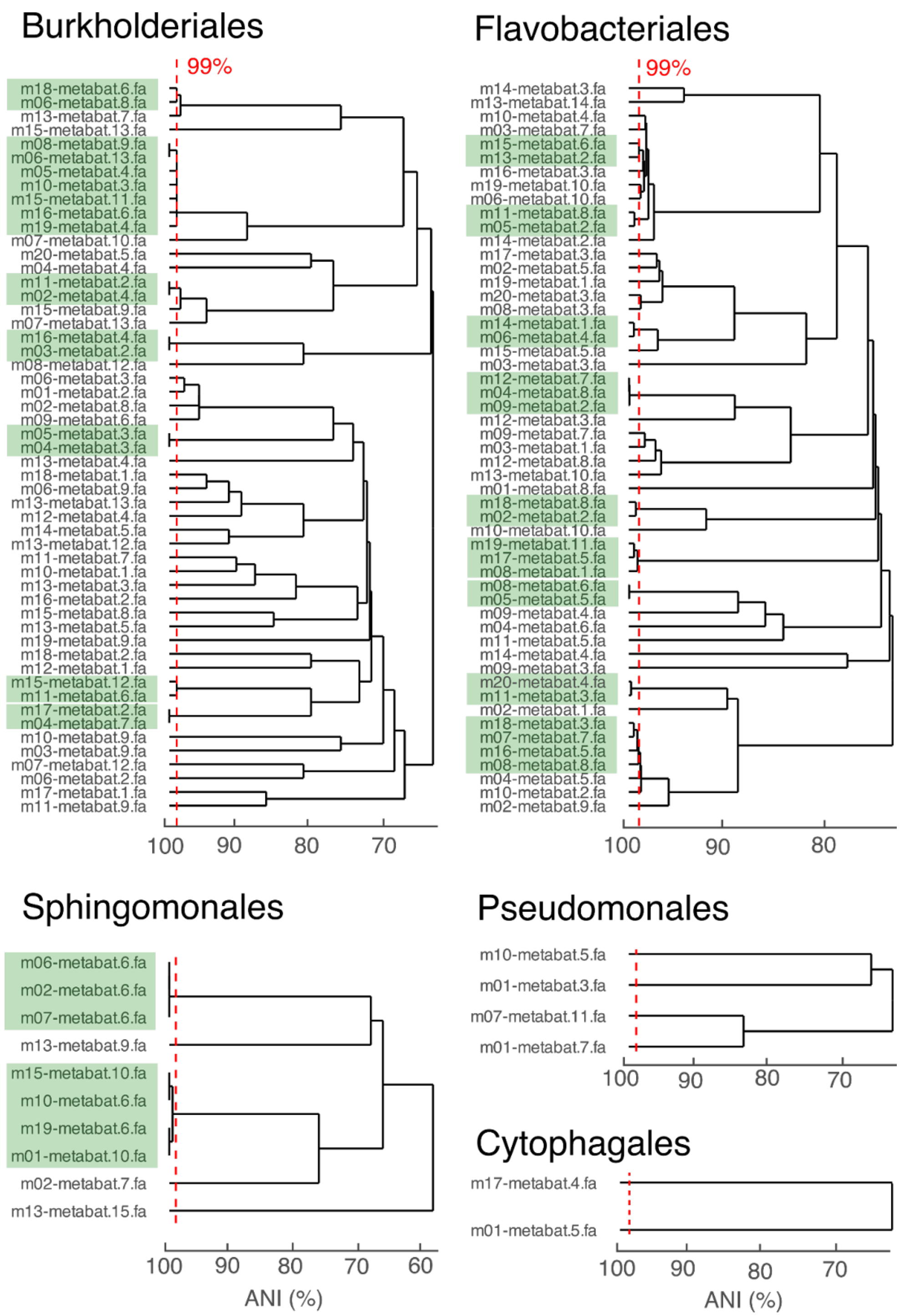

Fig S1: MAGs with an Average Nucleotide Identity (ANI) ≥ 99% across the communities were considered as identical populations and termed pooled MAGs (pMAGs, highlighted in green). For each taxonomic order, Hierarchical Agglomerative Clustering with average linkage was computed on the ANI.

Table S1 : Description of the genes and their KO identifiers involved in the glucose and cellobiose transport and degradation that were searched in addition to search pattern "glycolysis" and "Pentose Phosphate Pathway" across the datasets. The last column corresponds to the number of genomes in which the genes were found. The 6 genes we focused on are bolded.

	Genes	Description	KO identifiers	n MAGs (122)
**Glucose Transporters**				
	GtsA, GtsB, GtsC, malK	Glucose/mannose ABC	K17315, K17316, K17317, K10112	35
	GlcS, GlcU, GlcT, GlcV	Glucose/arabinose ABC	K10196, K10197, K10198, K10199	0
	MalE, MalF, MalG, MalK/MsmX	Maltose/Maltodextrin ABC	K10108, K10109, K10110, K10111/K10112	33
	MglB, MglC, MglA	Methyl-galactoside ABC	K10540, K10541, K10542	0
	PtsG, Crr	Glucose PTS	K02779, K02777	4
	PtsG	Glucose PTS	K20118	0
	MalX, Crr	Maltose/Glucose PTS	K02791,K02777	0
	NagE	N-Acetyl-D-glucosamine	K02804	7
	ManY, ManZ, ManXa/ManX	Mannose PTS	K02795, K02796, K02793/K02794	0
	galP	MFS, galactose: H+ symporter	K08137	0
	glcP	MFS, glucose/mannose H+ symporter	K08174	20
	glf	MFS, glucose uniporter	K13854	0
	mfsA	MFS, multidrug H+ antiporter	K07552	98
	glcU	glucose uptake protein	K05340	1
**Cellobiose metabolism**				
	*cebE, **cebF**, cebG, MsmX*	cellobiose ABC	K10240, **K10241**, K10242, K10112	3
	***celB**, celC, celA*	cellobiose PTS	**K02761**, K02759, K02760	5
	bglF/ bglFa	β-glucoside PTS	K02757/K02755	0
	AcsF, Crr	β-glucoside PTS	K02753, K02777	0
	E2.4.1.20	Phosphorylase	**K00702**	4
	Cbe, mbe	Epimerase	**K16213**	25
	bglU	β-glucosidase	K01188	0
	bglB	β-glucosidase	**K05350**	32
	bglA	6-phospho-β-glucosidase	K01223	0
	bglX	β-glucosidase	**K05349**	78
	CDH	Cellobiose dehydrogenase	K19069	0
	bglH	Carbohydrate-specific outer membrane porin	K10124	0
	celF	6-phospho-β-glucosidase	K01222	0

Table S2: Description of the 10 functional gene categories analysed, with the specific searched pattern in the description provided in the KEGG database.

Functional category	Searched pattern	Description
Signalling	"signal transduction" "secretion system"	two component systems, Phosphatidylinositol signalling system, stress proteins, MAPK signalling pathway
Regulation	"transcription factors" "transcriptional regulator"	27 families of transcription factors (LysR, TetR...), heat and cold repressors, Helix-turn-helix (HTH)
Transporters	"transporter"	ABC transport system, MFS transporters, SMR family and other ion-coupled transporters
Secondary Metabolites	"secondary metabolites"	biosynthesis and biodegradation of secondary metabolites
Amino-acids	"amino acid"	biosynthesis and biodegradation of amino-acids
Cofactors and vitamins	"cofactors and vitamins"	biosynthesis and biodegradation of cofactors and vitamins
Genetic information	"genetic information processing" **NOT** "signal transduction", "transcription factors" "transcriptional regulator"	Enzymes needed for replication, DNA repair and recombination, translation proteins, sigma factors, chaperones and heat shock proteins, RNA degradation, tRNAs, proteins involved in cell division
Glucose and cellobiose metabolism (Substrates)	"pentose phosphate" "glycolysis" + specific genes (see Supp. Table 1)	Proteins involved in the assimilation of glucose, glucose transporters and β-glucosidases
Carbohydrates metabolism	"carbohydrate" **NOT** in glucose and cellobiose metabolism	metabolism of other carbohydrates than glucose and cellobiose
Other metabolism	"metabolism" **NOT** in all other categories	Metabolism of lipids, proteins...

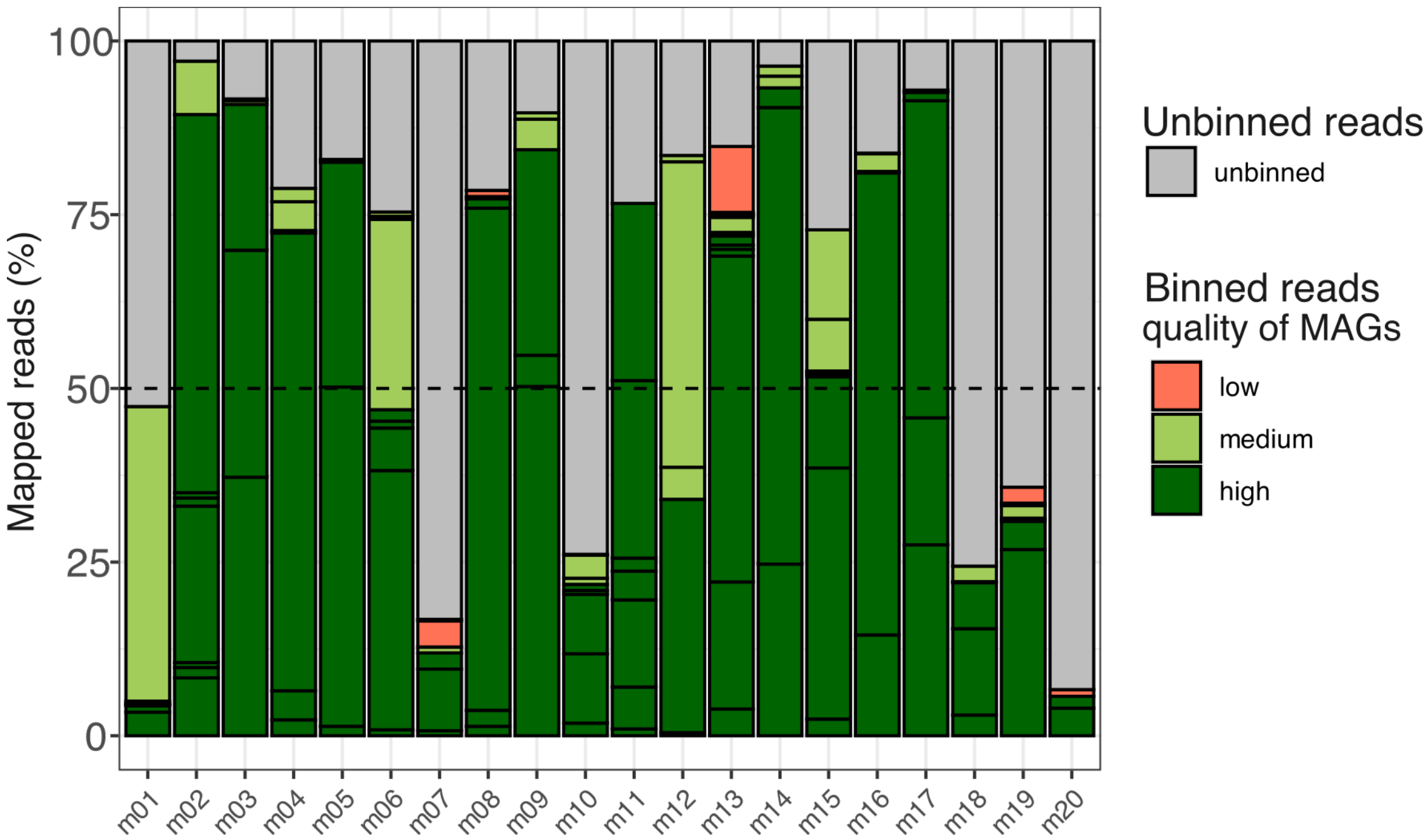

Fig S2: Proportions of the reads that were assigned to MAGs in each of the 20 communities. We considered that the MAGs were well representative of the communities and therefore suitable for a structure-function analysis when at least 50% of the reads could be binned into MAGs (dotted line). The MAGs were well representative of their community in 14 microcosms.The quality of the MAGs is displayed.

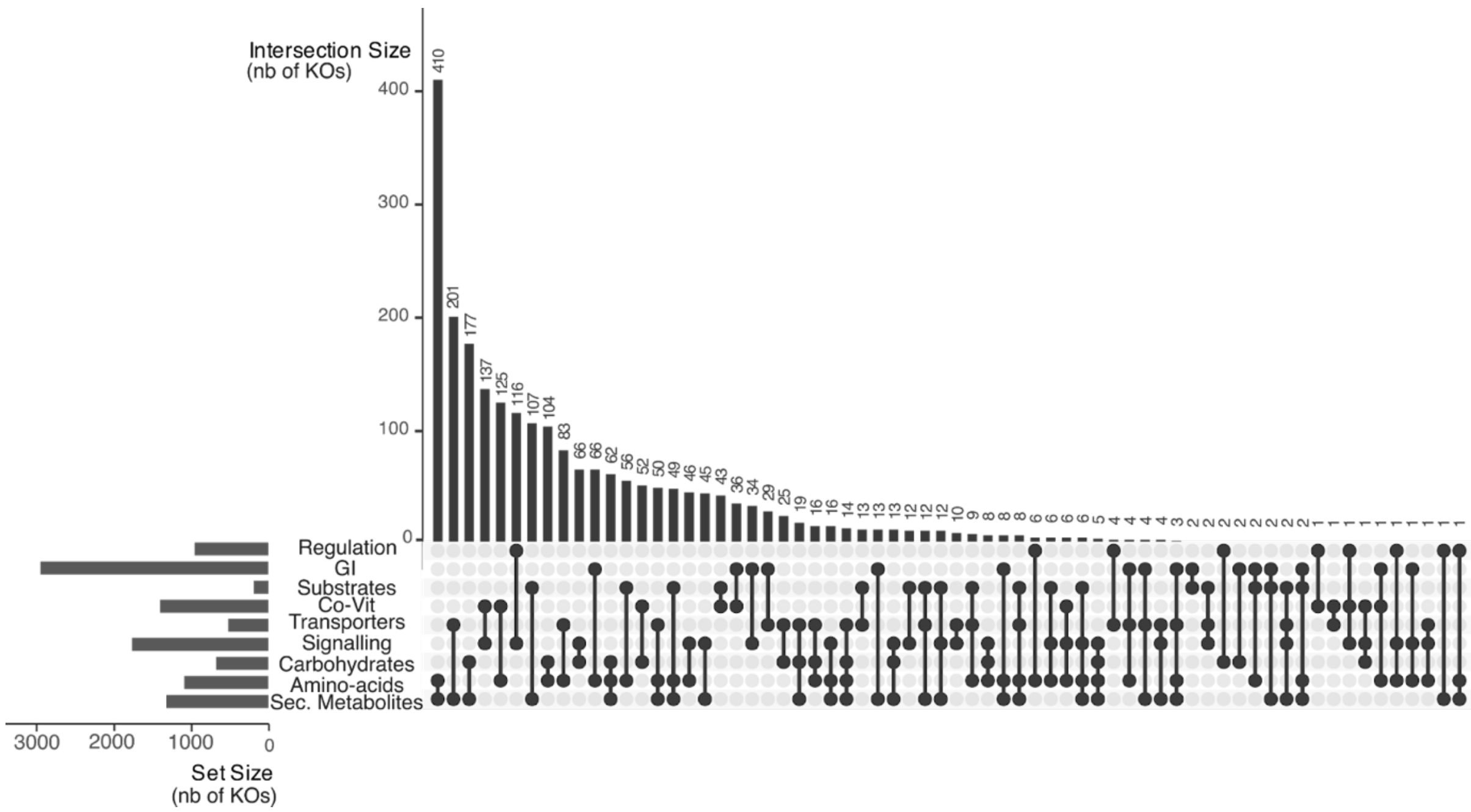

Fig S3: Upset plot of the intersections of categories, ie.e number of shared KOs. GI: Genetic Information, Co-Vit: Cofactors and Vitamins, Sec. Metabolites: Secondary Metabolites. The number of KOs per category is represented as the set size.

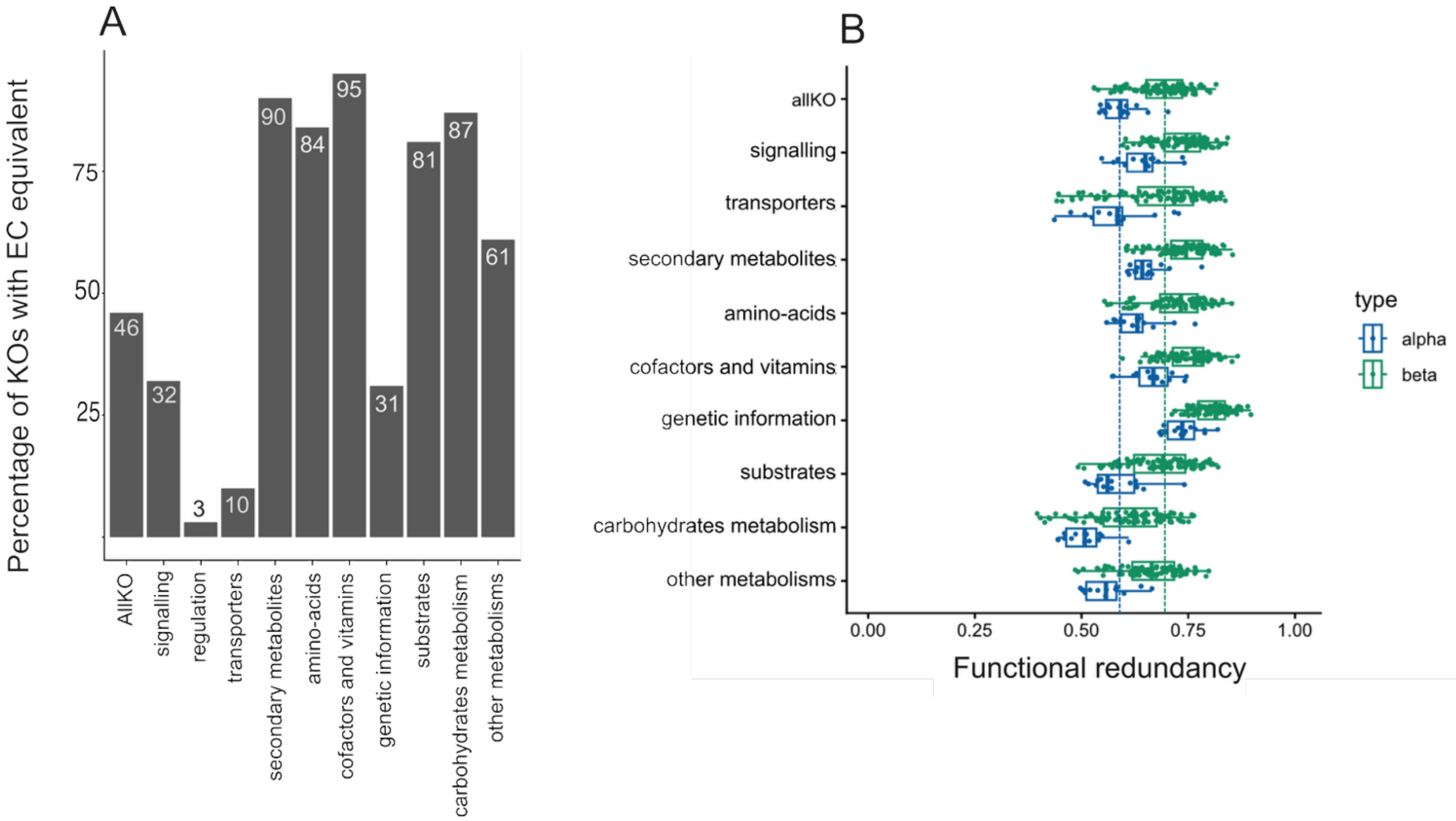

Fig S4: Complementary analysis of functional redundancy based on Enzyme Commission numbers (EC) instead of KOs **A**: Out of the 13 221 KOs from the KEGG database, 5644 have a corresponding complete EC number (42.6%) and this percentage varied greatly between the functional categories. **B**: α- and β-redundancy across all functional categories. The blue and green dotted lines represent the median α- and β-redundancy for the category "allKO" (i.e., all genes as EC numbers) respectively. The KOs from the category "regulation" had no equivalent EC number in the set of genes present in the microcosms (n=4495 for α-redundancy and n=4505 KOs for β-redundancy) . The pattern obtained was similar to the one obtained with KOs (Fig 3C), yet slight differences for the categories "signalling" and "transporters" were found. In these categories, only a small portion of KOs had EC equivalent (signalling=32% and transporters=10%), thus we are not able to conclude if measuring redundancy on EC numbers gave a clearer insight than the analysis on KOs, which appeared more comprehensive.

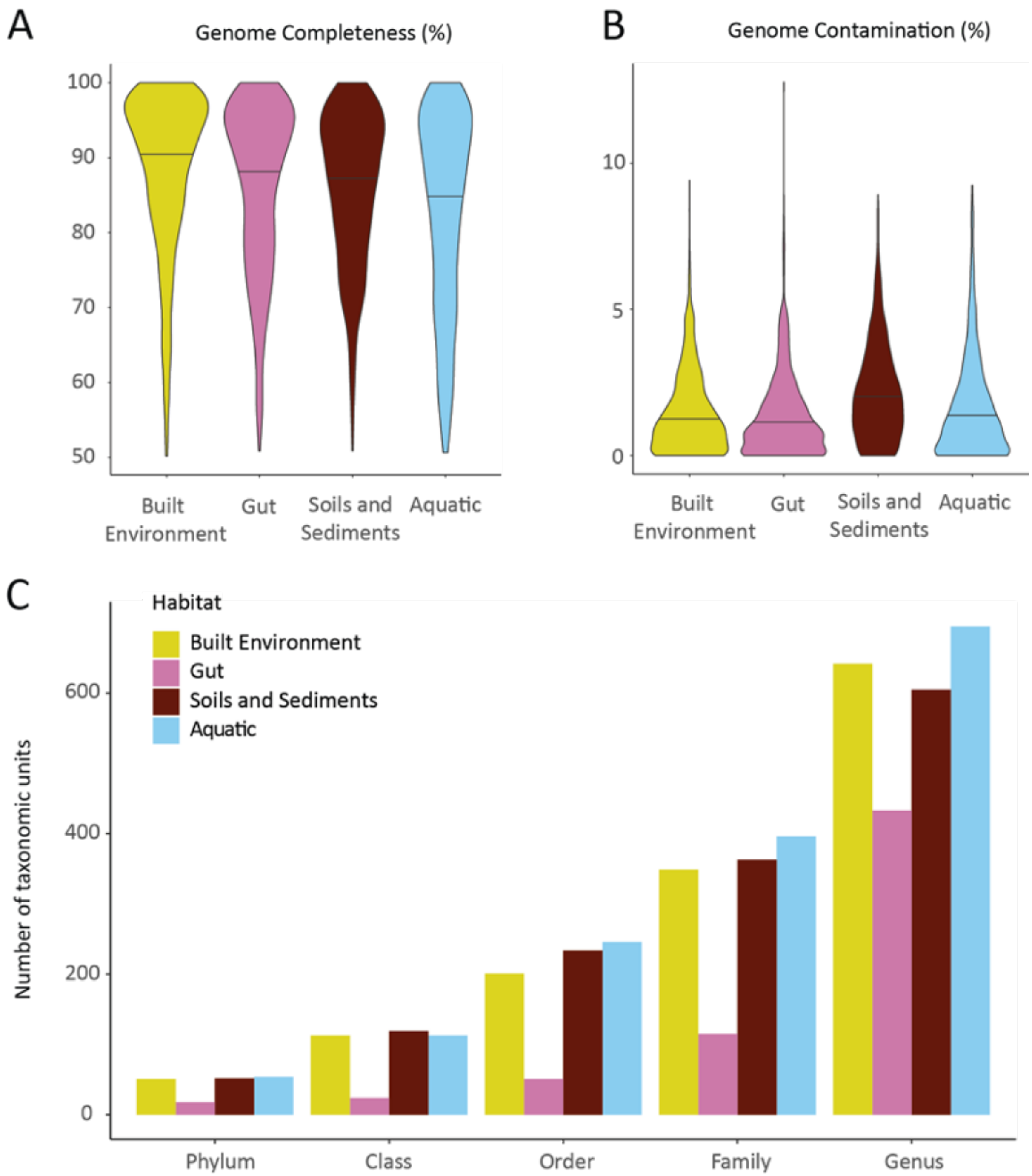

Fig S5: Description of genomes from 4 habitat types. Quality of the 3624 genomes (906 x 4) from the GTDB for each habitat **A**: genome completeness, **B**: genome contamination and **C**: Number of taxonomic units at different taxonomic levels among the 906 genomes of each habitat.

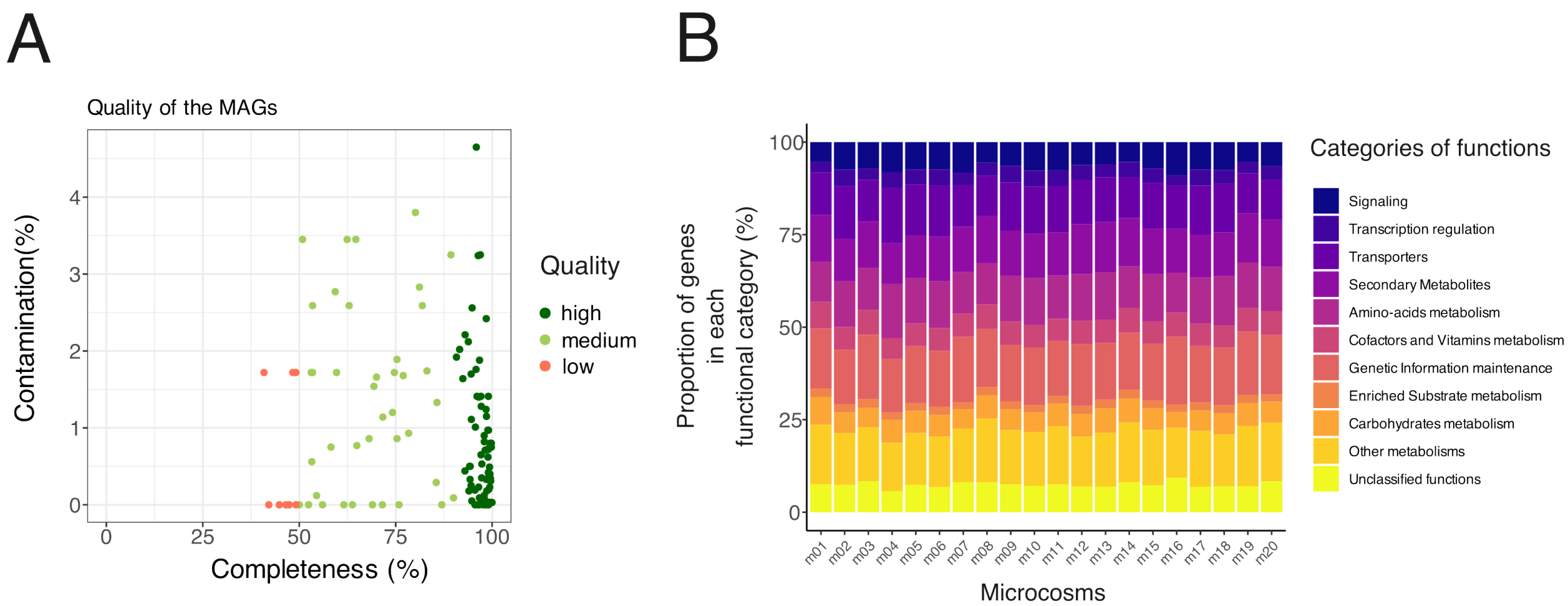

Fig S6: Description of the Metagenomes Assembled Genomes (MAGs) recovered from a stochastic community assembly experiment (Le Moigne *et al.*, 2023, Experiment 2). **A**: Quality of the 122 MAGs recovered in 20 communities. **B**: Genome-centric functional profile of each community. The genes of the MAGs were classified according to 10 major categories.

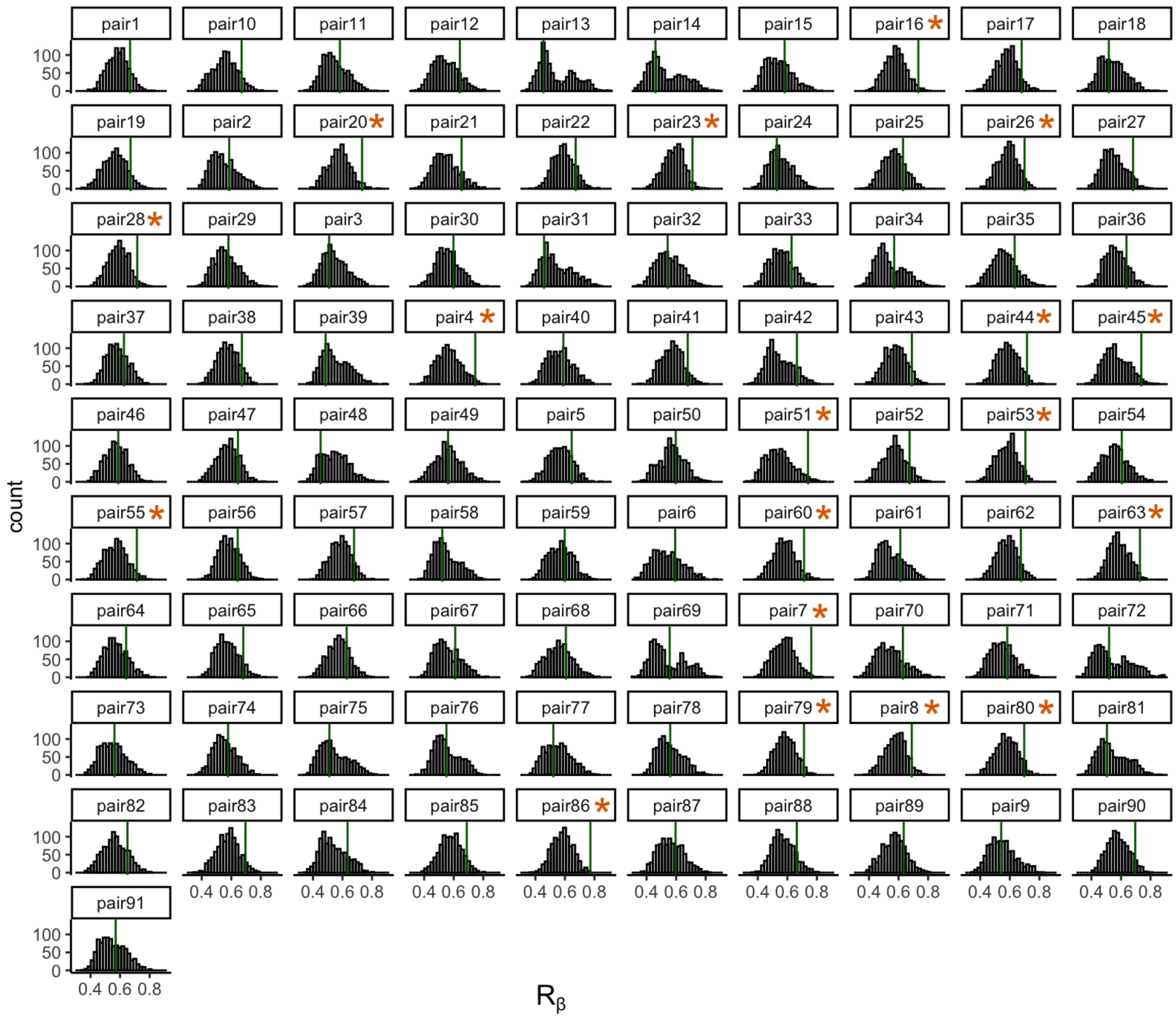

Fig S7: Null model (Grey histogram) and observed value (green line) of β- functional redundancy. The identity of the MAGs was randomised 999 times. Pairs of communities that were significantly different from the null model are indicated with an orange *.

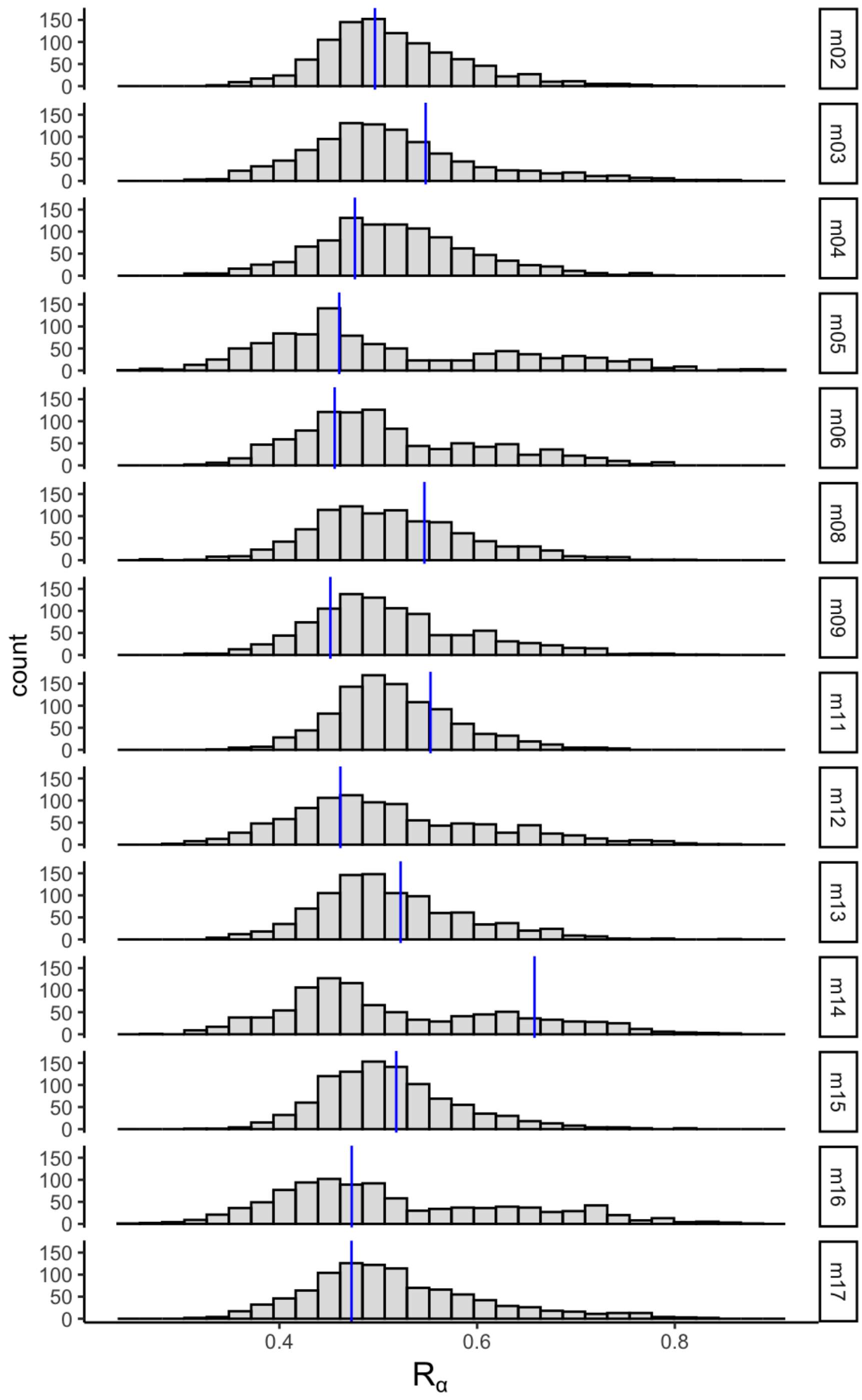

Fig S8: Null model (Grey histogram) and observed value (blue line) of α- functional redundancy. The identity of the MAGs was randomised 999 times. None of the 14 communities had an observed value significantly different from the null model.

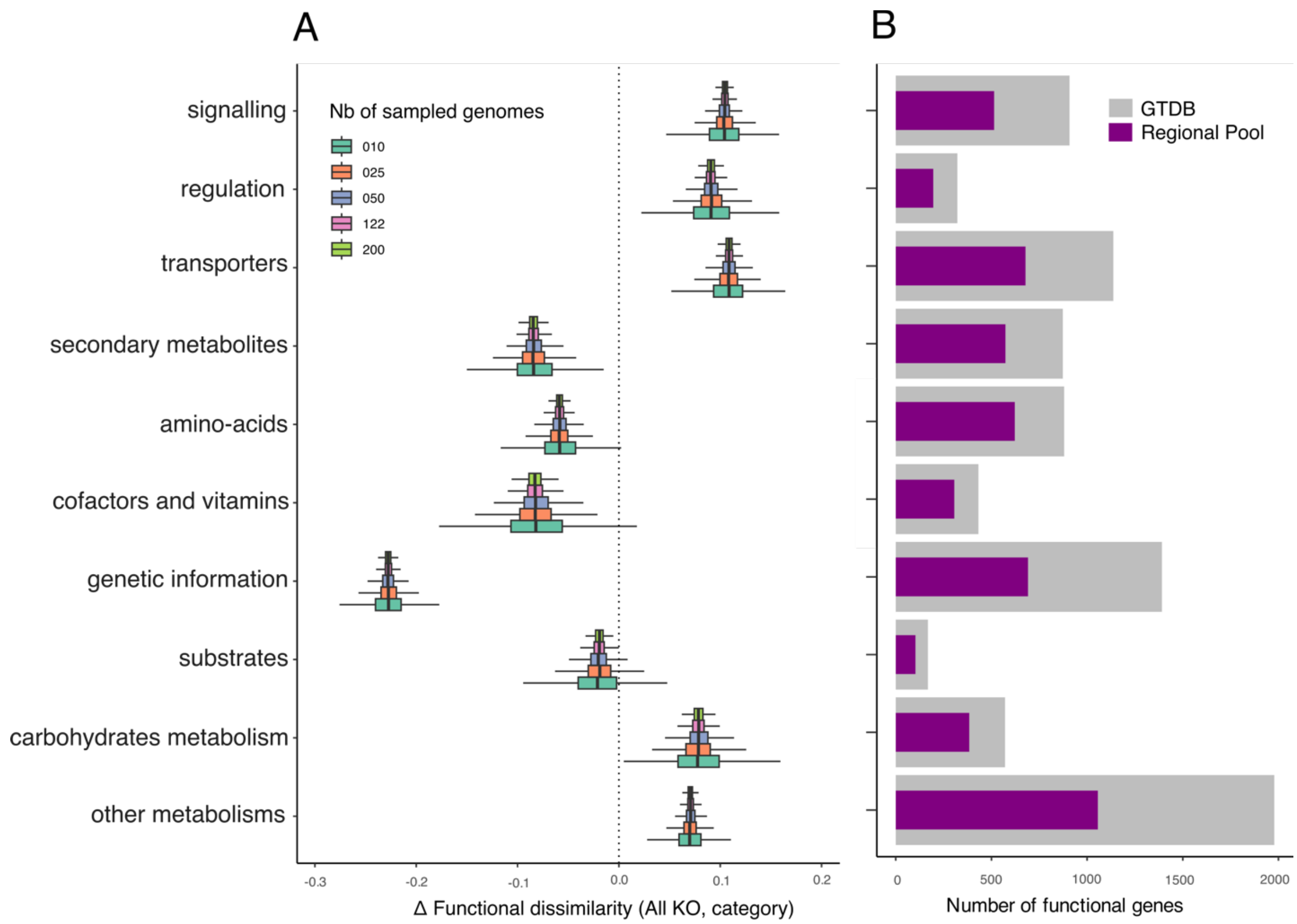

Betaregression Model:

**Variable**	**df**	χ^2	**p-value**
Functional Categegory	9	$2.57\ 10^{6}$	10^{-16}
Nb of sampled genomes	4	0.55	0.93

Fig S9: A: The number of randomly sampled genomes does not influence the observed pattern of functional dissimilarity (Genomes from the GTDB). We randomly subsampled a various number of genomes 999 times (n=10, n=25, n=50, n=122 and n=200) from the 28 714 x 8 342 taxa-by-traits matrix from the GTDB and examined the median functional dissimilarity (Bray-Curtis) across the categories. We calculated the difference between the median value and the "All KOs" category for each randomisation and for each category (Δ functional dissimilarity = category - All KOs). Differences in Δ functional dissimilarity across categories and the number of genomes in the set was evaluated with a beta regression (function "betareg" from the package "betareg") suitable for values confined to]0,1[. The data were thus rescaled to this interval by adding 1 to the original Δ functional dissimilarity and then dividing by 2. Post-hoc tests were performed using Estimated Marginal Means (EMMs) calculated with the function "emmeans" from the package "emmeans". The p-values were adjusted with Tukey's method to correct for multiple comparisons. The detailed results of the statistical analyses are in the Supp. Table 3. **B:** Comparison of the number of functional genes in each category between the MAGs from the experiment and the genomes from the GTDB. The number of genes per category does not influence the observed pattern.

References:

Le Moigne A, Randegger F, Gupta A, Petchey OL, Pernthaler J. Stochasticity causes high β-diversity and functional divergence of bacterial assemblages in closed systems. 2023;104(4):e4005. https://doi.org/10.1002/ecy.4005
